# Different approaches for bladder neck dissection during robot-assisted radical prostatectomy: the Aalst technique

**DOI:** 10.1590/S1677-5538.IBJU.2023.0027

**Published:** 2023-03-31

**Authors:** Carlo A. Bravi, Angelo Mottaran, Luca Sarchi, Adele Piro, Marco Paciotti, Luigi Nocera, Eleonora Balestrazzi, Maria Peraire, Rui Farinha, Kim Pauwaert, Manoe Van Herwaarden, Marie-Hélène Vinckier, Pieter De Backer, Frederiek D'Hondt, Ruben De Groote, Geert De Naeyer, Alexandre Mottrie

**Affiliations:** 1 Onze-Lieve-Vrouwziekenhuis Hospital Department of Urology Aalst Belgium Department of Urology, Onze-Lieve-Vrouwziekenhuis Hospital, Aalst, Belgium; 2 ORSI Academy Ghent Belgium ORSI Academy, Ghent, Belgium; 3 IRCCS Ospedale San Raffaele Unit of Urology Division of Oncology Milan Italy Division of Oncology/Unit of Urology; URI; IRCCS Ospedale San Raffaele, Milan, Italy; 4 IRCCS Azienda Ospedaliero-Universitaria di Bologna Division of Urology Bologna Italy Division of Urology, IRCCS Azienda Ospedaliero-Universitaria di Bologna, Bologna, Italy; 5 University of Modena and Reggio Emilia Department of Urology Modena Italy Department of Urology, University of Modena and Reggio Emilia, Modena, Italy; 6 IRCCS Humanitas Research Hospital Department of Urology Milan Rozzano Italy Department of Urology, Humanitas Research Hospital, IRCCS, Rozzano, Milan, Italy

## Abstract

**Introduction:**

Bladder neck dissection is one of the most delicate surgical steps of robotic-assisted radical prostatectomy (RARP) [1, 2], and it may affect surgical margins rate and functional outcomes [3, 4]. Given the relationship between outcomes and surgical experience [5–7], it is crucial to implement a step-by-step approach for each surgical step of the procedure, especially in the most challenging part of the intervention. In this video compilation, we described the techniques for bladder neck dissection utilized at OLV Hospital (Aalst, Belgium).

**Surgical Technique:**

We illustrated five different techniques for bladder neck dissection during RARP. The anterior technique tackles the bladder neck from above until the urethral catheter is visualized, and then the dissection is completed posteriorly. The lateral and postero-lateral approaches involve the identification of a weakness point at the prostate-vesical junction and aim to develop the posterior plane – virtually until the seminal vesicles – prior to the opening of the urethra anteriorly. Finally, we described our techniques for bladder neck dissection in more challenging cases such as in patients with bulky middle lobes and prior surgery for benign prostatic hyperplasia. All approaches follow anatomic landmarks to minimize positive surgical margins and aim to preserve the bladder neck in order to promote optimal functional recovery. All procedures were performed with DaVinci robotic platforms using a 3-instruments configuration (scissors, fenestrated bipolar, and needle driver). As standard protocol at our Institution, urinary catheter was removed on postoperative day two [8].

**Conclusions:**

Five different approaches for bladder neck dissection during RARP were described in this video compilation. We believe that the technical details provided here might be of help for clinicians who are starting their practice with this surgical intervention.
